# Fatal Fat Embolism Syndrome After Posterior Spinal Fusion: A Case Report

**DOI:** 10.7759/cureus.71480

**Published:** 2024-10-14

**Authors:** Andrew Park, Lancelot A Benn, Addisu Mesfin

**Affiliations:** 1 Orthopedic Surgery, University of Rochester, Rochester, USA; 2 Orthopedic Surgery, MedStar Washington Hospital Center, Washington, DC, USA

**Keywords:** bmp-2, bone grafting, fatal, fat embolism syndrome, lumbar stenosis, posterior spinal fusion

## Abstract

Posterior spinal fusion with instrumentation is a routine elective treatment of lumbar stenosis with radiculopathy and myelopathy. Fat embolism syndrome (FES) is a rare complication of this procedure. We describe the first documented case of fatal FES after an L3-L5 posterior spinal fusion using off-label bone morphogenic protein 2 (BMP-2) and allograft instead of iliac crest bone grafting. Postoperatively, the patient developed delayed cerebral symptoms with swift respiratory failure and cardiac arrest. Based on our investigation, it is likely that pedicle screw fixation was the cause of the fat embolisms with little effect from BMP-2.

## Introduction

Fat embolism syndrome (FES) is an uncommon but well-documented complication of orthopedic surgeries. It is historically characterized by acute respiratory failure, altered mental status, and a petechial rash, with marked variation in clinical presentation [1]. FES is primarily associated with trauma as well as intramedullary and joint procedures involving long bones [2,3]. Although studies have found microscopic fat emboli present in 90% of these cases, symptoms of FES were observed in only 0.5-23% of patients, with a mortality rate of 10-20% [2]. Few cases of FES after spinal surgery have been reported in the literature. Most have focused on younger patients undergoing scoliosis surgery or following vertebroplasty [1,4-11]. There are multiple case studies regarding fat embolism after posterior spinal surgery [12-16]. We present a unique case of a fatal pulmonary and systemic fat embolism where symptoms started on day 6 post-op after posterior spinal fusion using off-label bone morphogenic protein 2 (BMP-2) and allograft without iliac crest bone graft harvesting.

## Case presentation

**Figure 1:**
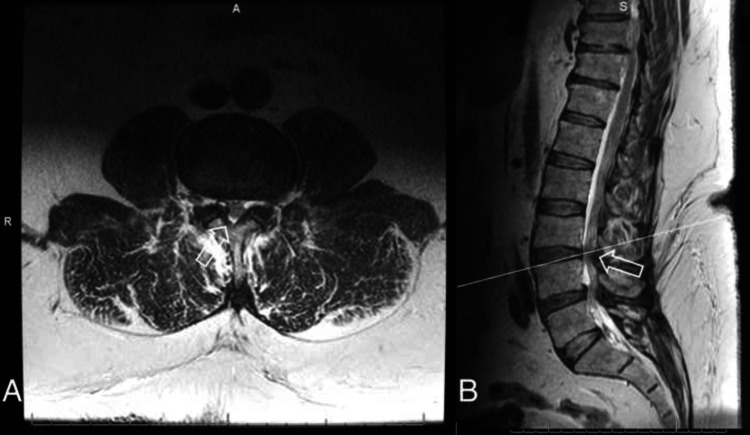
(A) Axial and (B) sagittal T2-weighted MRI of the lumbar spine demonstrating severe spinal stenosis at L3-L4 (arrows).

**Figure 2:**
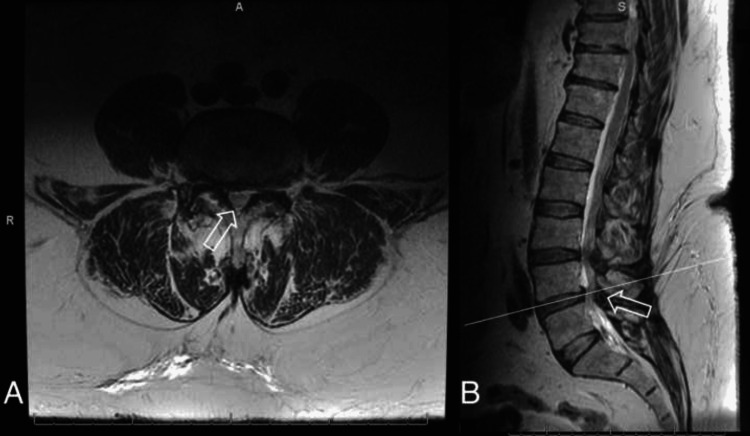
(A) Axial and (B) sagittal T2-weighted MRI of the lumbar spine demonstrating severe spinal stenosis at L4-L5 (arrows).

**Figure 3:**
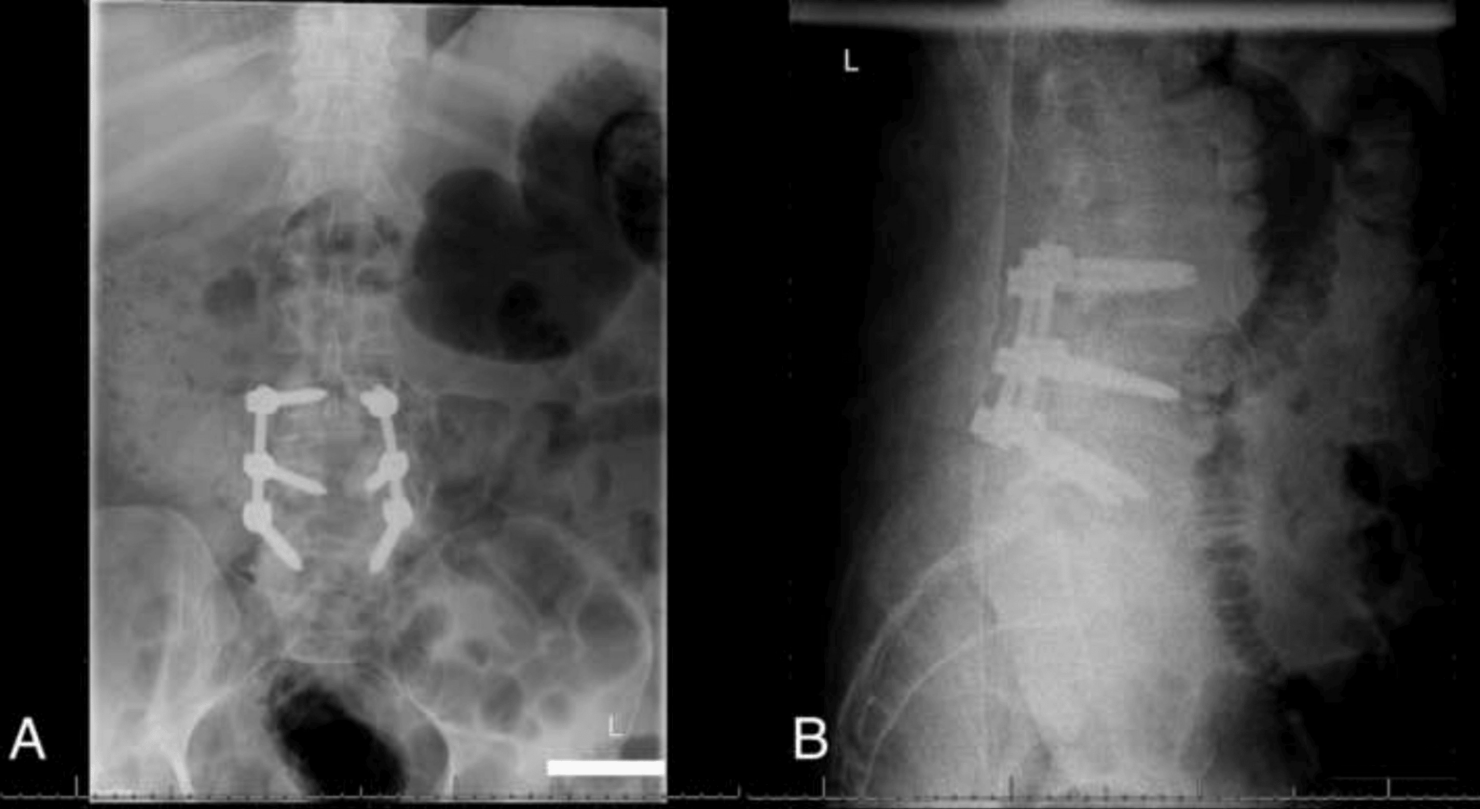
(A) Anteroposterior and (B) lateral radiograph of the lumbar spine demonstrating proper instrumentation alignment of L3-L5 posterior instrumentation.

A 62-year-old male presented to our clinic with a longstanding history of lower back pain with associated radicular and neurogenic claudication symptoms. He had a complicated history, including coronary artery disease (CAD) status post inferior ST-segment elevation myocardial infarction (STEMI) and percutaneous coronary intervention (PCI), ischemic cardiomyopathy with automated implantable cardioverter defibrillator (AICD), hypertension, hyperlipidemia, multiple sclerosis, type 2 diabetes, adrenal insufficiency on steroids, carotid stenosis, paroxysmal atrial fibrillation, history of right cerebellar cerebral vascular accident (CVA), neck surgery, obstructive sleep apnea (OSA), seizure disorder, and chronic daily hallucinations. He was one year status post C5-C7 anterior cervical discectomy fusion with significant resolution of cervical myelopathic symptoms. However, his lumbar symptoms persisted despite conservative treatment, such as physical therapy and steroid injections. Preoperative imaging studies demonstrated severe L3-L5 lumbar stenosis (see Figure 1 and Figure 2). The decision was made for surgical intervention. The patient underwent an L3-L5 posterior spinal instrumented fusion with L3-L5 laminectomy. Instead of the iliac crest bone graft, off-label BMP-2, local autograft, and allograft were used. Off-label BMP-2 was used to increase the fusion rate and avoid surgical time associated with iliac crest harvest in a patient with multiple medical comorbidities. There were no surgical complications. Postoperative imaging confirmed proper hardware alignment (see Figure 3).

On postoperative day 6, the patient developed worsening hallucinations, agitation, and physically aggressive behavior towards the staff. Hospital medicine, neurology, and psychiatry teams evaluated him for hallucinations and aggressiveness. A spot electroencephalogram (EEG) was negative for seizures. Given his history of chronic hallucinations, neurology recommended no immediate treatment and an expedited discharge.

**Figure 4:**
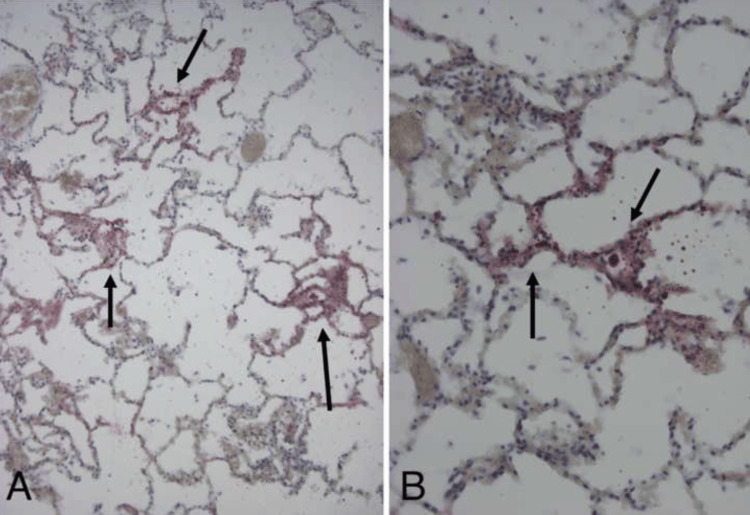
(A and B) ORO stains of the lung showing numerous ORO(+) fat globules in microvasculature and alveolar cell cytoplasm (arrows). ORO: Oil Red O

**Figure 5:**
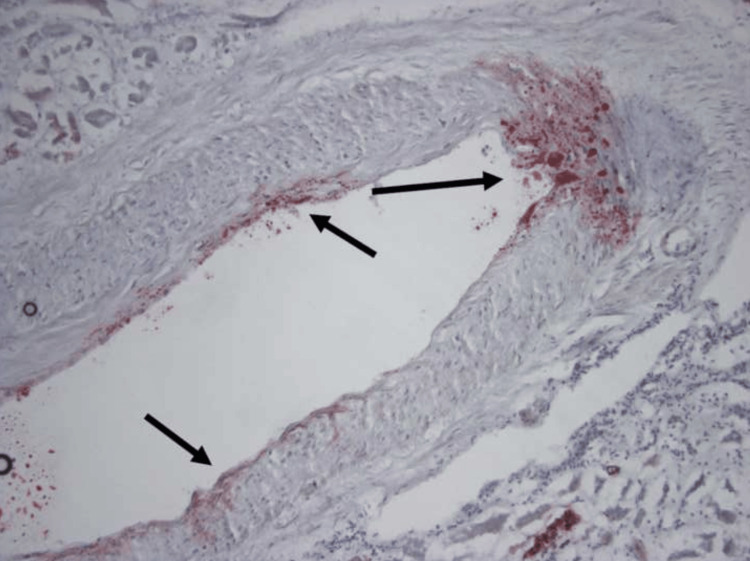
ORO stain of the kidney showing segmental intramural deposition of ORO(+) globules in intraparenchymal arteries (arrows). ORO: Oil Red O

On postoperative day 14, the nurses noted an acute change in his respiratory status. He became non-responsive, and they began compression. The code team responded for attempted resuscitation. The patient was in asystole upon examination. The patient was intubated, two intraosseous (IOs) drug administrations were done in bilateral lower extremities, and three rounds of epinephrine and 2 g of magnesium were administered without return of spontaneous circulation (ROSC). An autopsy revealed fat deposition in the microvasculature and alveolar cell cytoplasm of the lung, in addition to deposition in the intraparenchymal arteries of the kidney (see Figure 4 and Figure 5). Secondary cardiomyopathy with signs of biventricular heart failure and shock was also noted.

## Discussion

The literature describes two main hypotheses concerning the pathogenesis of FES. The mechanical theory postulates that increased intramedullary pressure drives large fat droplets out from the injured bone marrow. The venous system then transports them to the lungs. Once in the lung parenchyma, they mechanically obstruct the end capillary beds [1,6,8,9,17]. Some fat droplets may pass through the lungs, enter the systemic circulation, and cause embolization in other organs, such as the brain, retina, skin, or, in our case, kidney [8].

The biomechanical theory has two categories: obstructive and toxic [8]. The obstructive theory states that injury initiates a conglomeration of pre-existing chylomicrons through plasma mediators. The globules then enter the circulatory system and cause pulmonary vascular obstruction [6,8,17]. The toxic theory states that trauma and subsequently released fat particles precipitate an inflammatory response. This response causes hypercoagulability, vasoactive mediator release, and fatty acid precipitation. The inflammatory state augments the fat globules' embolic effect and affects the lungs' pneumocytes. This sequence creates a delayed clinical presentation, as in our patient, similar to acute respiratory distress syndrome [1,6,8].

There is no universal definition of FES. Gurd proposed the most commonly used criteria in 1970. Table 1 illustrates the major and minor features. Gurd stated that clinical diagnosis required at least one major feature plus four minor features [5]. However, no consensus in the literature exists regarding the accuracy of these criteria or the number of features needed for diagnosis. Our case satisfied the major features portion of the criteria, but we did not detect many of the minor features due to a rapid decline in clinical status. We diagnosed our patient with FES postmortem with autopsy findings of fat emboli in the lungs and kidneys and clinical presentation of acute respiratory failure and subsequent cardiac arrest.

**Table 1:** Major and minor features of fat embolism syndrome. ESR: erythrocyte sedimentation rate

Major features	Minor features
Petechial rash	Tachycardia
Respiratory symptoms plus bilateral signs with positive radiographic changes	Pyrexia
Cerebral signs unrelated to head injury or any other condition	Retinal changes (fat or petechiae)
	Urinary changes (anuria, oliguria, fat globules)
	A sudden drop in hemoglobin level
	Sudden thrombocytopenia
	High ESR
	Fat globules in the sputum

Takahashi et al. published a study analyzing 60 adult patients undergoing posterior lumbar surgery using intraoperative transesophageal echocardiography. Forty underwent laminectomies with posterior fusion with instrumentation. Twenty underwent lumbar surgery with laminectomies without instrumentation with or without discectomy. Twenty-three underwent harvesting of iliac bone grafts. They assessed each portion of the surgery for embolic events, and each embolic was categorized using a grading scale from Pitto et al. [9]. Grade 0 was defined as no emboli; grade 1 was a few fine emboli; grade 2 was a cascade of fine emboli or embolic masses with a diameter not exceeding 5 mm; and grade 3 was defined as emboli with a diameter of more than 5 mm. They found that 80% of instrumented patients had grade 2 or 3 embolic events compared to zero non-instrumented patients. They also found that the insertion of pedicle screws was the biggest inciting event for grade 2 or 3 embolic events. Conversely, surgical approach, laminectomy, disc removal, and bone harvesting were associated with a small number of emboli [12]. This study aligns with our findings, as we did not harvest iliac crest bone graft in our fusion [12,18].

Brandt et al. dispute this finding in their case report of a fatal pulmonary fat embolism after posterior spinal fusion with bilateral iliac crest bone grafts. They concluded that the fat embolism was possibly due to extensive harvesting of spongy bone, which may have collected in the femoral or iliac veins due to compression while the patient was prone. When the patient was transferred to the supine position, the buildup of fat emboli may have been released, initiating the fatal pulmonary embolism. They doubted that the embolism was due to transpedicular instrumentation [2].

In addition to the lack of iliac crest harvesting and a two-week delay in clinical presentation, the use of off-label BMP-2 makes this case unique. Esmail et al. published a review of complications associated with human recombinant bone morphogenic protein 2 (rhBMP2) in 5051 Medicare patients undergoing posterior/posterolateral lumbar fusion. Although they found a higher overall complication rate, the authors found no significantly higher risk of pulmonary embolism or respiratory complications in patients with rhBMP2 versus no rhBMP2 [3,19,20]. In addition to this study, we believe that the use of BMP-2 did not significantly contribute to our patient's demise as it was not found on autopsy as an obstructive agent.

There were limitations to this study. It is unclear whether our patient's aggression and hallucinations on postoperative day 6 were the presenting symptoms of FES or if it was due to his chronic underlying diagnosis or worsening delirium due to extended hospital stay. In a study of 100 patients with FES after orthopedic trauma, Gurd and Wilson found 34 patients initially presented with cerebral symptoms. The most common symptoms were confusion and drowsiness [6]. An EEG was negative for seizures, and the consulting team did not think his symptoms warranted brain imaging. In addition, the typical ischemic and petechial hemorrhagic white matter lesions were not detected on autopsy, so the likelihood that imaging would have aided in an earlier diagnosis is low.

## Conclusions

FES is a dangerous complication of orthopedic surgeries and trauma. We presented a case of fatal FES after posterior lumbar instrumentation and fusion and the use of off-label BMP-2 without iliac crest bone grafting. Based on our results, we had a high index of suspicion that pedicle screw fixation was possibly the cause of the pulmonary and systemic embolisms.
